# Whole-exome tumor sequencing study in biliary cancer patients with a response to MEK inhibitors

**DOI:** 10.18632/oncotarget.6632

**Published:** 2015-12-16

**Authors:** Daniel H. Ahn, Hatice Gulcin Ozer, Baris Hancioglu, Gregory B. Lesinski, Cynthia Timmers, Tanios Bekaii-Saab

**Affiliations:** ^1^ Department of Internal Medicine, Divison of Medical Oncology, Ohio State University Comprehensive Cancer Center, Columbus, OH, USA; ^2^ Department of Biomedical Informatics, Ohio State University, Columbus, OH, USA

**Keywords:** biliary cancer, whole-exome sequencing, MEK inhibition

## Abstract

We previously conducted a phase-II study with selumetinib (AZD6244), a small molecule inhibitor of MEK1/2, in advanced biliary tract cancers (BTC), where the primary endpoint was response rate. Several patients experienced objective response. These findings were confirmed with MEK162 in a similar patient population. To assess for tumor-specific genetic variants that mediate sensitivity to *MEK* inhibition in BTC, we performed whole-exome sequencing in patients with an objective response to selumetinib. Normal and tumor DNA from FFPE tissue from two patients who experienced an objective response underwent whole-exome sequencing. Raw sequence reads were processed with GATK workflow and tumor specific variants were identified using MuTect and VarScan2. Ensemble Variant Effect Predictor was used to determine functional consequences of these variants. Copy number changes and potential gene fusion events were also screened. Findings were compared to assess for any commonality between the two tumor samples, and whether the identified variants were intrinsic to the *MAPK* pathway. 1169 and 628 tumor-specific variants were identified in the two samples. Further analysis demonstrated 60 and 53 functional and novel variants, respectively. Of the identified tumor-specific variants, fusion events or copy number changes, no commonality was seen. Several variants in genes associated with *ERK* signaling were present in each tumor sample. Although there were no common tumor-specific variants in the two patients who exhibited an objective response to selumetinib, several genes associated with *ERK* signaling were identified. Confirmatory studies investigating the role of the identified genes and other potential tumor independent factors need further investigation.

## INTRODUCTION

Biliary tract cancers (BTC) comprise of malignancies of the intrahepatic, extrahepatic bile ducts and the gallbladder. The disease is rare, where less than 15,000 cases are diagnosed in the United States each year [1]. Surgical approaches, including resection or liver transplantation are the only curative treatment approaches for BTC [2]. Unfortunately, most patients present with advanced disease at the time of diagnosis. The current standard regimen for untreated advanced BTC is a combination of cytotoxic chemotherapy with gemcitabine and cisplatin, and the disease remains universally fatal, with a median survival that remains less than one year [3]. The poor outcomes with first-line treatment and absence of approved therapies in the refractory setting highlight the need to develop new and more effective treatments for biliary cancers [4-6].

Pre-clinical studies demonstrated genomic alterations in downstream signaling pathway activation and led to an increased interest in investigating novel targeted agents in clinical trials for BTC. Therapies aimed at inhibiting targets in signaling pathways have demonstrated promising pre-clinical activity aimed at *MEK/ERK*, the downstream effector the mitogen activated protein kinase (*MAPK*) pathway. The initial rationale for *MEK* inhibition was based on the reported high prevalence of activated *RAS* signaling pathways in biliary cancer [7], with subsequent activation of downstream signaling pathways, including *MAPK*. Additionally, *BRAF* mutations were shown to be frequently associated with a more sensitive phenotype to *MEK* inhibition and constitute a survival mechanism for mutant cells [7]. *BRAF* mutations were identified in up to 22% of human biliary cancer samples in one study, which were mutually exclusive of KRAS mutations [8], providing further support to *MEK* inhibition as a rational therapeutic target in BTC.

With the above rationale, we initiated and completed phase II study of selumetinib (AZD6244; AstraZeneca, Manchester, UK), a second-generation, potent, selective and uncompetitive small molecule inhibitor of MAP kinase, MEK1/2, in advanced or metastatic BTC. The primary endpoint of the study was response rate, which was measured according to the Response Evaluation Criteria in Solid Tumors (RECIST) 1.0, assessed by computed tomography (CT) or magnetic resonance imaging (MRI) every 8 weeks [9]. Selumetinib showed preliminary promising activity with a 12% objective response rate (3 partial response) and a 68% disease control rate [10, 11]. Of the three patients who experienced an objective clinical response, 1 patient demonstrated a reduction in tumor marker CA 19-9 by 60%, while the other two patients were non CA 19-9 secretors. Samples from all 28 patients underwent limited genotyping for *MEK* relevant targets, *BRAF* and *KRAS*. We did not identify any *BRAF* V600E mutations in this patient cohort [11]. Less than 10% of patients exhibited *KRAS* mutations that did not correlate with a meaningful response. An expanded analysis that included *KRAS, BRAF, NRAS, PIK3CA and PTEN* in the follow up study with MEK162 in BTC again found no correlation with response [12].

To better understand potential drivers that mediate sensitivity to *MEK* inhibition in BTC, we conducted a full comprehensive analysis utilizing whole exome sequencing to assess for tumor-specific variants in patients who experienced a response to selumetinib.

## RESULTS

**Figure 1:**
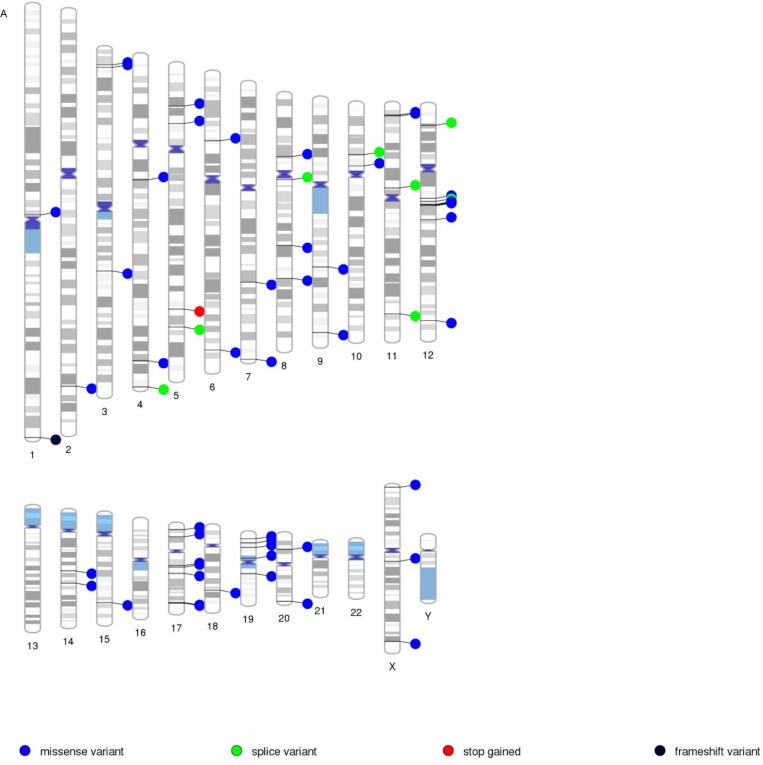

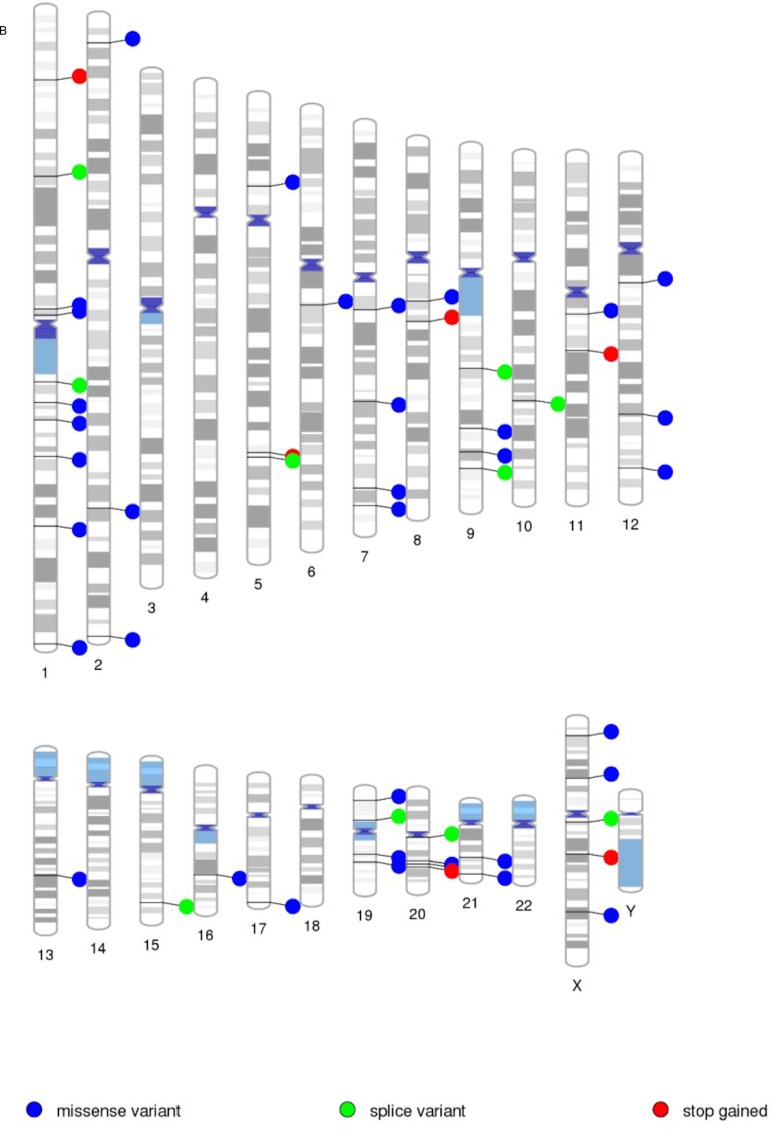
Visualization of novel and functional variants across the genome Tumor specific variants (missense, splice, stop/gained and frame shift) are presented with PenoGram for tumor sample one (A) and two (B) respectively. **A.** Tumor Sample 1. **B.** Tumor Sample 2.

Whole exome sequencing of the patients’ tumor and normal sample pairs were conducted. Tumor specific variants, i.e. somatic point mutations, somatic INDELs, and loss of heterozygosity (LOH) events, were identified. Table 1 lists number of such events and tools used to detect them. We then used Ensembl VEP to determine the effect of the identified variants on genes, transcripts and protein sequence, as well as regulatory regions. We filtered the findings first by identifying all tumor-specific variants, and second, by eliminating any variants found in the dbSNP and ESP databases. We retained all novel variants (those not found in dbSNP or ESP), frame shift and stop gain variants and missense variants with a severe functional consequence (either deleterious by SIFT, or probably/possibly damaging by PolyPhen). As a result, we identified 60 and 53 tumor specific novel and functional variants in sample one and two respectively (Table 2). Figure 1 depicts genome-wide locations of these variants as a PhenoGram [12]. Tumor specific novel and functional variants were analyzed through QIAGEN's Ingenuity^®^ Pathway Analysis (IPA^®^, QIAGEN Redwood City,www.qiagen.com/ingenuity) to assess whether any variants were intrinsic to the RAS/RAF/MAPK pathway (Supplementary Table 3).

**Table 1:** Number of somatic variants and LOH events in tumor samples

Samples	Somatic Point Mutations			Somatic INDEL	LOH (Point Mutations)	LOH (INDEL)
	MuTect	MuTect & VarScan	VarScan Somatic	VarScan	VarScan	VarScan
1	206	68	72	18	733	72
2	238	3	81	24	250	32

We did not identify any commonality in tumor-specific variants (novel and functional) between the two tumor samples or gene variants specific to the RAS/RAF/MAPK pathway (Supplementary Table 1, Supplementary Table 2). However, several of the identified genes such as NACA, and CUL2 and their associated pathways are linked to *MAPK* signaling [13-15]. In addition to somatic variants, we examined the two tumor samples displayed for any concordance in gene fusion and copy number events. Acknowledging the limitations of detecting gene fusion events with whole exome sequencing data, we looked for any fusion events of the tumor/normal pairs from our data. Using in house scripts we extracted discordant reads from the alignments and examined potential gene fusion events. We did not detect any fusion events.

Lastly, utilizing VarScan 2, we assessed whether any common changes in copy number were seen between both patient samples. We selected copy number variation (CNV) based on at least 1.5x fold-change in copy number in a given candidate. Copy number changes were found in chromosomes 1, 5, 6, 9, 10, 12, 17, 20, 21 and 22 in one sample, while the second tumor sample had copy number changes in chromosome 11 and 12 (Supplementary Table 3, Supplementary Table 4). We did not identify any similarities in copy number changes and its genes between the two tumor samples.

**Table 2:** Number of somatic variants in tumor samples and their classification

	Sample 1	Sample 2
Tumor specific variants	1169	628
Novel variants	524	383
Functional and novel variants	60	53

## DISCUSSION

Biliary tract cancer (BTC) is a rare cancer and is universally lethal, which highlights the urgency to develop novel therapies. The findings from our previous studies consistently suggest that a subset of patients will develop a meaningful objective response to *MEK* inhibitors [10, 11]. Our initial limited “targeted” analyses of *PTEN*, *KRAS*, *BRAF*, *PI3KCA* and *MET* did not elucidate predictors of response to *MEK* inhibitors with any tumor-specific genetic variants. In order to further assess for tumor-specific genetic variants that mediate sensitivity to *MEK* inhibition in BTC, we performed whole-exome sequencing in patients with an objective response to single-agent selumetinib.

These additional studies failed to identify any common tumor-specific variants including somatic variants, fusion events and copy number analyses in the examined tumor samples. While the pathway analysis did not demonstrate any of the variants to be genes directly involved with the *MAPK* pathway, several of the identified functional genomic variants including NACA, and CUL2 and their associated pathways are linked to *MAPK* signaling [13-15]. N-acetylcysteine amide, the protein encoded by NACA, interacts with *ERK* signaling and is believed to have an anti-apoptotic effect on cells [16]. CUL2 and its protein product are integrated with hypoxia-inducible factors (HIFs) and are shown to be upregulated by *MAPK* signaling [17]. Additionally, CUL2 has been identified in other malignancies as a predictor for clinical response to chemotherapy [15]. While limited, these interesting findings are novel and worthy of further exploration in prospective studies to assess their potential predictive role for *MEK* inhibition in BTC.

The absence of definitive genomic variants that may predict for response to *MEK* inhibition suggest that possible alternative variants or mechanisms may be responsible for the clinical activity consistently observed with MEK inhibitors across 2 studies. It is possible, although unlikely, that expanded whole genome analysis may reveal additional structural variants (such as large insertion/deletions, tandem duplications, gene fusions) that were not identified in this analysis. Alternatively, the oncogenic “driver” may be independent of the tumor itself but rather related to tumor extrinsic factors. Pre-clinical studies suggest a role for activated *MAPK* pathway as a pro-angiogenic driver and/or as an immunomodulatory effector [18-20]. A phase II randomized study investigating the use of the MEK inhibitor tramenitib vs. a fluoropyrimidine in patients with refractory BTC (SWOG 1310; ClinicalTrials.gov NCT02042443), has incorporated an expanded cytokine and immune cell analysis to confirm the above hypothesis. Finally, the *MAPK* pathway has been shown to influence epigenetic “drivers” of biliary tract carcinogenesis and future DNA methylation analysis may reveal patterns that would explain the selective clinical response to *MEK* inhibition in biliary cancer [21-23].

The results from our analysis may be limited by the use of DNA derived from FFPE verses fresh frozen samples. However, a next-generation sequencing study of 16 paired fresh-frozen and FFPE tumor samples demonstrated that formalin fixation results in smaller NGS library insert sizes, greater coverage variability and an increase in C to T transitions. [24]. In that study, error rate, library complexity and coverage statistics were not significantly different. Comparison of base calls between paired samples showed a high rate of concordance (> 99%) with 97% agreement in detected single-nucleotide variants and > 98% accuracy in collected data in comparison to genotypes from a single-nucleotide polymorphism array platform [24]. These findings along with others demonstrate that the differences between FFPE and fresh tissue are not significant and FFPE samples can be reliably used for whole exome sequencing by NGS [25-27].

Additionally, the use of adjacent normal tissue as germline control is not ideal since we cannot rule out position effect in the data. Moreover, the inclusion of tumor samples from patients that progressed on therapy may have been further informative, however, given the sample size and lack of correlation in the tumor specific variants between the two responders, this may not proved to be useful. To alleviate these limitations, moving forward, we are collecting fresh biopsies when appropriate and whole blood samples for use as a germline control.

In conclusion, our comprehensive analysis through whole-exome sequencing did not identify common tumor-specific changes in patients who responded to *MEK* inhibition in BTC. Although we did not identify common tumor-specific somatic changes in the two patients who exhibited an objective response to selumetinib, several genes associated with *ERK* signaling were identified. Confirmatory studies, with the prospective collection of fresh, unfixed tissue and blood samples for germline genetic analysis, investigating the role of the identified genes and other potential tumor independent factors need to be further investigated. An improved understanding of molecular and immunomodulatory abnormalities is key for developing a platform for screening patients for molecularly guided basket clinical trials with available targeted therapies including *MEK, FGFR* and other inhibitors with promising activity in BTC [7, 28, 29].

## MATERIALS AND METHODS

The protocol of this study were reviewed and approved by the Ohio State University Institutional Review Board. The methods were carried out in accordance with the approved guidelines and regulations. All the patients provided written informed consent before the initiation of the study. Eligible patients who were included in the initial phase II study were all required to have histologically confirmed advanced biliary tract carcinoma, up to 1 prior systemic anti-cancer therapy, no prior exposure to *MEK* or *RAF* inhibitors, and with measurable disease per Response Evaluation Criteria in Solid Tumors (RECIST) [9, 10]. Eligible patients were required to have histologically confirmed biliary tract carcinoma that was surgically unresectable. All patients were required to have either fresh or paraffin-embedded tissue from tumor blocks prior to enrolling onto the study. Radiologic assessment was done by computed tomography or magnetic resonance imaging every 8 weeks, and responses were measured according to RECIST. Three out of twenty-eight patients (12%) had a confirmed objective response, and 2 of those patients [patient number #14 and 20] had adequate tissue sample to send for further DNA sequencing [10].

### DNA sequencing

DNA was extracted from surgically resected formalin-fixed paraffin-embedded tissue (FFPE) using the Maxwell 16 FFPE DNA purification kit (Promega, Inc) and quantitated using pico green on a Qubit (Life Technologies). For tumor tissue, an H&E stained section was marked by a pathologist and tumor containing regions were macro-dissected from serial sections to enrich for tumor content. As a blood sample was not available, germline DNA was derived from FFPE blocks containing adjacent normal tissue. Whole exome capture libraries were constructed from 250 ng of DNA and sequenced by the Broad Institute Genomics Platform on an Illumina HiSeq 2500. The average number of QC>30 reads was 75 million for the normal DNA samples and 250 million reads for the tumor DNA samples with coverage of 68% and 76% targeted bases at >20X respectively.

### Data analysis

Raw sequence reads were processed and aligned to human reference genome at the Broad Institute using the Genome Analysis Toolkit (GATK) workflow, a software to analyze high-throughput sequencing data, with a primary focus on variant discovery and genotyping, and several measures to ensure data quality assurance [30]. We utilized two well-established tools, MuTect (v1.1.4) and VarScan 2 (v2.3.7), to identify tumor-specific variants for each two patient sample. MuTect is a method developed at the Broad Institute (Cambridge, MA) to identify somatic point mutations in next-generation sequencing (NGS) data of cancer genomes [31]. VarScan2, a platform-independent software tool (developed at Washington University; Saint Louis, Missouri), detects variants (somatic point mutations, insertion/deletions and loss of heterozygosity events) in NGS data [32, 33]. A recent review suggests that MuTect and VarScan 2 detect most variants than any other tools [34]. Therefore, we chose to utilize both tools to confirm the findings of each tool and to complement each test given the limitation of MuTect's ability to only detect somatic mutations. Finally, we used Ensembl Variant Effect Predictor (VEP) to annotate and determine functional consequences of tumor specific variants [35]. VarScan 2 was also used to identify copy number changes in tumor samples compared to matching normal samples. Potential gene fusion events were screened using a series of in-house shell and R scripts.

## SUPPLEMENTARY TABLES








